# Clinical prognostic indicators of dysphagia following prolonged orotracheal intubation in ICU patients

**DOI:** 10.1186/cc13069

**Published:** 2013-10-18

**Authors:** Danielle Pedroni Moraes, Fernanda Chiarion Sassi, Laura Davison Mangilli, Bruno Zilberstein, Claudia Regina Furquim de Andrade

**Affiliations:** 1 Hospital das Clínicas, School of Medicine, University of São Paulo, Rua Ovídeo Pires de Campos, 186, Cerqueira César, CEP 054030-010 São Paulo, SP, Brazil; 2 Department of Physiotherapy, Speech-Language and Hearing Sciences, and Occupational Therapy, School of Medicine, University of São Paulo, Rua Ovídeo Pires de Campos, 186, Cerqueira César, CEP 054030-010 São Paulo, SP, Brazil; 3 Medical Investigation Laboratory (LIM 34) - Rehabilitation Sciences, School of Medicine, University of São Paulo, Rua Ovídeo Pires de Campos, 186, Cerqueira César, CEP 054030-010 São Paulo, SP, Brazil; 4 Digestive Surgery Division, School of Medicine, University of São Paulo, Rua Ovídeo Pires de Campos, 186, Cerqueira César, CEP 054030-010 São Paulo, SP, Brazil

## Abstract

**Introduction:**

The development of postextubation swallowing dysfunction is well documented in the literature with high prevalence in most studies. However, there are relatively few studies with specific outcomes that focus on the follow-up of these patients until hospital discharge. The purpose of our study was to determine prognostic indicators of dysphagia in ICU patients submitted to prolonged orotracheal intubation (OTI).

**Methods:**

We conducted a retrospective, observational cohort study from 2010 to 2012 of all patients over 18 years of age admitted to a university hospital ICU who were submitted to prolonged OTI and subsequently received a bedside swallow evaluation (BSE) by a speech pathologist. The prognostic factors analyzed included dysphagia severity rate at the initial swallowing assessment and at hospital discharge, age, time to initiate oral feeding, amount of individual treatment, number of orotracheal intubations, intubation time and length of hospital stay.

**Results:**

After we excluded patients with neurologic diseases, tracheostomy, esophageal dysphagia and those who were submitted to surgical procedures involving the head and neck, our study sample size was 148 patients. The logistic regression model was used to examine the relationships between independent variables. In the univariate analyses, we found that statistically significant prognostic indicators of dysphagia included dysphagia severity rate at the initial swallowing assessment, time to initiate oral feeding and amount of individual treatment. In the multivariate analysis, we found that dysphagia severity rate at the initial swallowing assessment remained associated with good treatment outcomes.

**Conclusions:**

Studies of prognostic indicators in different populations with dysphagia can contribute to the design of more effective procedures when evaluating, treating, and monitoring individuals with this type of disorder. Additionally, this study stresses the importance of the initial assessment ratings.

## Introduction

The clinical relevance of dysphagia after extubation is profound; it occurs frequently and affects patients across all medical and surgical diagnostic categories [1,2]. For many hospitalized, intubated patients, underlying conditions may interact with dysphagia to produce aspiration, pneumonia, and/or respiratory compromise. Dysphagia even without aspiration can interfere with nutrition and delay clinical recovery [2].

Prolonged intubation, typically defined as longer than 48 hours [1,3-6], is thought to contribute to swallowing dysfunction. The development of postextubation swallowing dysfunction is well documented in the literature with high prevalence in most studies, ranging from 44 to 87% [7-11]. However, there are relatively few studies with specific outcomes that focus on the follow-up of these patients until hospital discharge. High-quality studies are relevant to assess the influence of prolonged intubation on dysphagia and to determine which factors put patients at greater risk [11].

Longer intubation duration has been correlated to dysphagic patients [7,12-14] and has also been reported to be an independent predictor of dysphagia severity [15,16]. The higher risk of dysphagia post extubation was reported in those patients with Glasgow Coma Scale scores of ≤14 [8] or age ≥55 yrs [8,16]. In contrast, another study found that neither age nor the duration of intubation was correlated with an increase in swallowing dysfunction in post orotracheal intubation (OTI) patients [1]. Post prolonged intubation swallowing disorder extends the time to begin the oral myofunctional/swallowing assessment, to return to normal oral feeding and delays subsequent hospital discharge [14,15,17].

When reviewing the literature, it appears that detailed analyses on the relationship between the degree and outcome of swallowing problems and the type and degree of primary treatment are very limited. A clearer understanding of prognostic indicators has the potential to enable the rehabilitation team to better predict recovery and facilitates appropriate and cost-effective care for individuals with swallowing disorders [18,19]. Studies have examined general prognostic indicators of swallowing function in different diseases. These indicators include: age [20,21]; severity of the disease [22-24]; cognitive status [18,25]; dysphagia severity level at hospital admission and/or discharge [22,26-28]; presence of feeding tube [29]; time to achieve oral feeding status [14,17,30]; time to tracheostomy weaning [14,31]; ventilator status [17,25]; amount of treatment [14,18,26]; and length of hospital stay [12,32].

Carefully selected process indicators should be used when evaluating the quality of the health care provided to patients with dysphagia. To enable a fair evaluation of clinical practice, process indicators should reflect expected practices in local settings, such as those found in relevant clinical guideline recommendations [19,33]. Many of the process indicators used previously for dysphagia management evaluations are not based on sound levels of evidence, which reflects the challenge of research in this field [33]. Therefore, the purpose of our study was to determine prognostic indicators related to dysphagia at hospital discharge of intensive care unit (ICU) patients submitted to prolonged orotracheal intubation.

## Materials and methods

Using the School of Medicine Hospital - University of São Paulo, Brazil - medical records system, we conducted a retrospective, observational cohort study of extubated ICU patients who had undergone a bedside swallow evaluation (BSE) by a speech pathologist. The project was approved by the Research Ethics Committee of the institution (*Comitê de Ética para Análise de Projetos de Pesquisa do HCFMUSP* Protocol number 0673/11). This study was approved as a retrospective review of documents without a requirement for patient consent.

### Patient population

Patients were eligible if they met all of the following criteria: (1) admission to any ICU during the two-year period from June 2010 to June 2012, (2) submitted to prolonged intubation (>48 hours), (3) BSE by a speech pathologist 24 to 48 hours after extubation, (4) older than 18 years of age, (5) clinical and respiratory stability and (6) more than 14 points on the Glasgow Coma Scale. Moreover, subjects were limited to those requiring assistance and presenting swallowing level ≤4 according to the American Speech-Language-Hearing Association National Outcome Measurement System (ASHA NOMS) [18,26,34]. Patients were excluded if they (1) were making use of a tracheostomy tube, (2) presented neurologic diseases, (3) presented esophageal dysphagia and (4) had been submitted to surgical procedures involving the head and neck.

Our hospital has as a standard procedure to refer all patients submitted to prolonged intubation to a BSE. However, only patients who are clinically stable undergo a complete BSE. Based on the existing literature [18], we included in our study patients who had initial poor swallowing levels. Although these patients were clinically stable, they still depended on alternative feeding methods or had moderate diet restriction. In our practice we have observed that patients who present good initial swallowing levels (ASHA NOMS swallowing levels 5 to 7) have functional swallowing and need little intervention and minimal cueing.

### Measurements - clinical assessment of swallowing

The BSE included the application of the Dysphagia Risk Evaluation Protocol (DREP) [35], followed by the classification of the swallowing functional level according to the ASHA NOMS [26,34].

The DREP [35] is a Brazilian bedside assessment protocol designed for the early detection of dysphagia risk. It includes the controlled offer of water and puree/solid volumes. The DREP determines whether the patient should receive larger volumes and different textures of food and liquids, and the amount of monitoring necessary for safe feeding. The protocol is divided into two sections - the water swallow test and the puree/solid swallow test - and results are marked as either pass or fail for each one of the observed items. As determined by the authors of the protocol, patients were assessed during the swallow of 5 ml of water offered on a syringe, 3, 5 and 10 ml of fruit puree offered on a spoon and half a piece of bread (the tests were repeated, if necessary, up to three times to confirm results). The assessment procedures consisted of 11 items for the water swallow test and 12 items for the puree/solid swallow test as presented in Table 1: patients were in the upright position, so that the position would not interfere in the results of the research.

**Table 1:** Definition of behavioral variables and oromotor test on the Dysphagia Risk Evaluation Protocol (DREP)

	**Variable**	**Clinician’s judgment**
**Water swallow test (5 ml)**	Extra oral loss	Water does not escape from the lips, manages bolus adequately - pass
		Difficulty in managing bolus, presents drooling/spillage from the mouth - fail
	Oral transit time	Swallows the bolus within 4 seconds - pass
		Takes longer than 4 seconds to swallow bolus or does not swallow - fail
	Nasal reflux	Water does not escape from the nasal cavities - pass
		Water comes out from the nasal cavities - fail
	Multiple swallows per bolus	Presents one swallow per bolus - pass
		Presents more than one swallow per bolus, presents drooling/spillage from the mouth, needs cues to complete the task - fail
	Laryngeal elevation (monitored by positioning the index and middle fingers over the hyoid bone and the thyroid cartilage)	Reaches an average elevation of two fingers of the examiner - pass
		Does not present laryngeal elevation or presents average elevation of less than two fingers of the examiner - fail
	Cervical auscultation (a stethoscope is placed at the lateral aspects above the cricoids cartilage in front of the sternocleidomastoid muscle and large vessels)	Presents the three characteristic sounds indicating that the bolus has gone through the pharynx - two clicks followed by an expiratory sound - pass
		Does not present any sound or sounds other than those described above - fail
	Oxygen saturation (baseline oxygen saturation registered prior to the swallowing test using a monitor or pulse oximetry)	Does not present changes in oxygen saturation in more than 4 units - pass
		Presents changes in oxygen saturation in more than 4 units - fail
	Voice quality	Does not present any alterations within the first minute after swallowing - pass
		Voice becomes gurgly (‘wet’) within the first minute after swallowing - fail
	Cough	Does not cough within the first minute after swallowing - pass
		Presence of cough (voluntary or not) followed or not by throat clearing within the first minute after swallowing - fail
	Choking	Does not choke after swallowing - pass
		Chokes during and/or after swallowing - fail
	Other signs (cardiac and respiratory frequencies)	Does not present significant changes in cardiac frequency (60–100 beats per minute) and in respiratory frequency (12–20 breaths per minute) - pass
		Presents signs of cyanoses, bronchospasm and significant alterations of the vital signs - fail
**Puree/solid swallow test (3, 5, 10 ml; half a piece of bread)**	Extra oral loss	Bolus does not escape from the lips, manages bolus adequately - pass
		Difficulty in managing the bolus, presents spillage from the mouth - fail
	Oral transit time	Swallows the bolus within 20 seconds - pass
		Takes longer than 20 seconds to swallow the bolus or does not swallow - fail
	Nasal reflux	The bolus does not escape from the nasal cavities - pass
		The bolus comes out from the nasal cavities - fail
	Oral residue	Presents absence or up to 25% of bolus residue in the oral cavity - pass
		Presents more than 25% of bolus residue in the oral cavity - fail
	Multiple swallows per bolus	Presents one to three swallows per bolus - pass
		Presents more than three swallows per bolus, presents drooling/spillage from the mouth, needs cues to complete task - fail
	Laryngeal elevation (monitored with the positioning of the index and middle fingers over the hyoid bone and the thyroid cartilage)	Reaches an average elevation of two fingers of the examiner - pass
		Does not present laryngeal elevation or presents average elevation of less than two fingers of the examiner - fail
	Cervical auscultation (a stethoscope is placed at the lateral aspects above the cricoids cartilage in front of the sternocleidomastoid muscle and large vessels)	Presents the three characteristic sounds indicating that the bolus has gone through the pharynx - two clicks followed by an expiratory sound - pass
		Does not present any sound or sounds other than those described above - fail
	Oxygen saturation (baseline oxygen saturation registered prior to the swallowing test using a monitor or pulse oximetry)	Does not present changes in oxygen saturation in more than 4 units - pass
		Presents changes in oxygen saturation in more than 4 units - fail
	Voice quality	Does not present any alterations within the first minute after swallowing - pass
		Voice becomes gurgly (‘wet’) within the first minute after swallowing - fail
	Cough	Does not cough within the first minute after swallowing - pass
		Presence of cough (voluntary or not) followed or not by throat clearing within the first minute after swallowing - fail
	Choking	Does not choke after swallowing - pass
		Chokes during and/or after swallowing - fail
	Other signs (cardiac and respiratory frequencies)	Does not present significant changes in cardiac frequency (60–100 beats per minute) and in respiratory frequency (12–20 breaths per minute) - pass
		Presents signs of cyanoses, bronchospasm and significant alterations of the vital signs - fail

The ASHA NOMS swallowing level scale is a multidimensional tool designed to measure both the supervision level required and diet level by assigning a single number between 1 to 7 (Table 2). For this study, the patients’ specific diet level and level of supervision required were used to assign the ASHA NOMS swallowing scale. Initial diet and supervision levels were documented at the first clinical evaluation and at dysphagia resolution/hospital discharge. The speech-language pathologist assigning the ASHA NOMS swallowing level had successfully passed specific training tests.

**Table 2:** American Speech-Language-Hearing Association National Outcome Measurement System (ASHA NOMS) swallowing level scale

Level 1	Individual is not able to swallow safely by mouth. All nutrition and hydration is received through non-oral means (for example nasogastric tube).
Level 2	Individual is not able to swallow safely by mouth for nutrition and hydration but may take some consistency with consistent maximal cues in therapy only. Alternative method of feeding is required.
Level 3	Alternative method of feeding is required as individual takes less than 50% of nutrition and hydration by mouth, and/or swallowing is safe with consistent use of moderate cues to use compensatory strategies and/or requires maximum diet restriction.
Level 4	Swallowing is safe but usually requires moderate cues to use compensatory strategies, and/or individual has moderate diet restriction and/or still requires tube feeding and/or oral supplements.
Level 5	Swallow is safe with minimal diet restriction and/or occasionally requires minimal cueing to use compensatory strategies. May occasionally self cue. All nutrition and hydration needs are met by mouth at mealtime.
Level 6	Swallowing is safe, and individual eats and drinks independently and may rarely require minimal cueing. Usually self cues when difficulty occurs. May need to avoid specific food items (for example popcorn and nuts), or requires additional time (due to dysphagia).
Level 7	Individual’s ability to eat independently is not limited by swallow function. Swallowing would be safe and efficient for all consistencies. Compensatory strategies are effectively used when needed.

All the patients received individual swallowing treatment until dysphagia resolution or hospital discharge. Patients in this study were assisted by various staff SLPs with experience in the area of dysphagia and trained to apply the same treatment program. The amount of treatment was recorded in revenue value units (RVUs) [18]. According to the literature, each RVU represents 15 minutes of actual therapy time.

### Prognostic indicators

All information regarding the swallowing treatment was registered in each patient’s file. Specific outcomes related to OTI were also recorded. The prognostic indicators selected for this study are aspects encompassed in the speech-language pathology scope of practice and are not formally reported by other members of the rehabilitation team [14,16-18,31]. The prognostic indicators included: dysphagia severity rate 1 (DSR1); dysphagia severity rate 2 (DSR2); time to initiate oral feeding (TOF); amount of individual treatment (RVU); number of orotracheal intubations (NOI); intubation time (IT); length of hospital stay (LS). Definitions of the prognostic indicators are presented in Table 3.

**Table 3:** Definition of prognostic indicators

	**Indicators**	**Definition**
**General**	Dysphagia severity rate 1 (DSR1)	ASHA NOMS swallowing level at initial swallowing assessment
	Dysphagia severity rate 2 (DSR2)	ASHA NOMS swallowing level at dysphagia resolution/hospital discharge
	Time to initiate oral feeding (TOF)	Time to start oral feeding after DSR1 (in days)
	Amount of individual treatment (revenue value unit (RVU))	Amount of individual swallowing treatment until dysphagia resolution/hospital discharge (in RVUs)
**Specific to the group of patients**	Number of orotracheal intubations (NOI)	Total number of orotracheal intubations
	Intubation time (IT)	Total duration of orotracheal intubation (in hours)
	Length of hospital stay (LS)	Time from hospital admission to discharge (in days)

### Data analysis

Analysis was performed using SPSS for Windows, version 13.0 (SPSS Inc., Chicago, IL, USA). For the present study, patients who had their swallowing classified as levels 6 or 7, according to the ASHA NOMS swallowing level scale, at dysphagia resolution/hospital discharge (DSR2) were considered as presenting good treatment outcomes. The proposed prognostic indicators were analyzed considering this goal. The purpose of this analysis was to identify which prognostic indicators were the most significant predictors of good treatment outcomes in the investigated population.

In order to show the overall results, variables were descriptively presented in contingency tables comprising absolute (n) and relative (%) frequencies. The logistic regression model was used to examine the relationships between independent variables. As previously described, the dependent variable was considered good treatment outcome (that is ASHA NOMS levels 6 and 7). The independent variables were: gender, age, DSR1, TOF, RVU, NOI, IT and LS. All variables were analyzed using the univariate model to determine significance (*P* ≤0.10). All significant variables and the interactions between them were used to obtain a selection for the multivariate model (*P* ≤0.05), according to the simultaneous entry procedure. The variables that remained in the model were independent prognostic variables. Spearman rank correlation coefficients examined any linear association among all prognostic indicators.

## Results

Of the 1,080 ICU patients who were referred to a BSE, 456 had been submitted to OTI, 85% (388) had records of prolonged OTI. Of the remaining patients, 148 met the inclusion criteria (91 males, mean age 53.51 ± 16.18; 57 females, mean age 52.88 ± 19.32). Table 4 shows the overall descriptive data.

**Table 4:** Descriptive data

**Variable**	**Mean**	**Median**	**SD**	**Min**	**Max**	**Q**		
						**25**	**50**	**75**
**AGE**	53.26	55	17.40	18	90	43	55	65
**LS**	43.07	34	30.96	9	197	21	34	58
**NOI**	1.08	1	0.27	1	2	1	1	1
**IT**	187.70	144	123.25	0	720	4	6	10
**TOF**	4.58	0	10.51	0	57	0	0	3
**RVU**	6.59	4	5.88	1.33	41.33	2.67	4	8

*SD*, Standard deviation; *Min*, Minimum; *Max*, Maximum; *Q*, Quartile; *LS*, Length of hospital stay; *NOI*, Number of orotracheal intubations; *IT*, Intubation time; *TOF*, Time to initiate oral feeding; *RVU*, Amount of individual treatment (revenue value unit).

Table 5 shows the distribution of the ASHA NOMS results on the initial swallowing assessment (DSR1) and at dysphagia resolution/hospital discharge (DSR2). As observed, most of the participants were classified as level 4 by the ASHA NOMS swallowing level scale on the initial swallowing assessment (that is individuals had moderate diet restriction and/or still required the use of a feeding tube). When looking at the distribution of participants among the different ASHA NOMS levels at the initial assessment and at dysphagia resolution/hospital discharge (DSR1 × DSR2), we can observe that 103 patients improved their swallowing to ASHA NOMS levels 5 to 7.

**Table 5:** Distribution of the American Speech-Language-Hearing Association National Outcome Measurement System (ASHA NOMS) levels at initial swallowing assessment (DSR1) and at discharge (DSR2)

	**ASHA NOMS levels at initial swallowing assessment**		**ASHA NOMS levels at discharge**	
**ASHA NOMS levels**	**N**	**%**	**N**	**%**
1. Not able to swallow by mouth	10	6.75	7	4.73
2. Takes some consistency with maximal cues	48	32.43	11	7.43
3. Takes less than 50% of nutrition by mouth with moderate cues	27	18.24	12	8.11
4. Swallowing is safe with moderate cues	63	42.56	15	10.14
5. Swallowing is safe with minimal cues	0	0	14	9.65
6. Swallowing is safe and rarely requires minimal cues	0	0	36	24.31
7. Swallowing is efficient, individual is independent	0	0	53	35.81

*DSR1*, Dysphagia severity rate 1; *DSR2*, Dysphagia severity rate 2; *N*, Number of patients; % percentage.

Table 6 shows the mean RVU obtained among the different levels on the ASHA NOMS scale at the initial assessment. The results indicate that the less severe the swallowing impairment, the lower the number of RVU.

**Table 6:** Mean revenue value units according to the dysphagia severity rate at the initial assessment

**DSR1 - ASHA NOMS level**	**N**	**RVU (mean)**
1. Not able to swallow by mouth	10	13.73
2. Takes some consistency with maximal cues	48	7.50
3. Takes less than 50% of nutrition by mouth with moderate cues	27	6.52
4. Swallowing is safe with moderate cues	63	4.80
5. Swallowing is safe with minimal cues	-	-
6. Swallowing is safe and rarely requires minimal cues	-	-
7. Swallowing is efficient, individual is independent.	-	-

*DSR1*, Dysphagia severity rate 1; *N*, Number of patients; *RVU*, Revenue value unit.

Univariate analyses performed to identify independent variables for good treatment outcomes in patients submitted to prolonged OTI are described in Table 7. Statistically significant prognostic indicators included ASHA NOMS at initial swallowing assessment (DSR1), time to initiate oral feeding (TOF) and amount of individual treatment (RVU). Multivariate logistical regression analysis (Table 8) was performed to determine whether the association between DSR1, TOF and RVU remained after the other indicators of good prognosis had been removed. In this analysis only DSR1 remained independently associated with good treatment outcomes.

**Table 7:** Logistic regression (univariate analysis) of the independent variables for good treatment outcomes (DSR2 levels 6/7)

	**OR**	***P *** **value**	**CI (95%)**
**DSR1**	2.294	0.001*	1.590 – 3.310
**Gender**	1.569	0.200	0.788 – 3.124
**Age**	0.989	0.253	0.970 – 1.008
**TOF**	0.960	0.025*	0.926 – 0.995
**RVU**	0.949	0.085*	0.894 – 1.007
**NOI**	0.922	0.894	0.278 – 3.055
**IT**	1.011	0.742	0.947 – 1.079
**LS**	0.998	0.665	0.987 – 1.008

*Significant result. *DSR2*, Dysphagia severity rate 2; *OR*, Odds ratio; *CI*, Confidence interval; *DSR1*, Dysphagia severity rate 1; *TOF*, Time to initiate oral feeding; *RVU*, Amount of individual treatment (revenue value unit); *NOI*, Number of orotracheal intubations; *IT*, Intubation time; *LS*, Length of hospital stay.

**Table 8:** Logistic regression (multivariate analysis) of the independent variables for good treatment outcomes (DSR2 levels 6/7)

	**OR**	***P *** **value**	**CI (95%)**
**DSR1**	1.547	0.050	0.999 – 2.396
**TOF**	0.986	0.560	0.942 – 1.033
**RVU**	0.964	0.394	0.885 – 1.049

*DSR2*, Dysphagia severity rate 2; *OR*, Odds ratio; *CI*, Confidence interval; *DSR1*, Dysphagia severity rate 1; *TOF*, Time to initiate oral feeding; *RVU*, Amount of individual treatment (revenue value unit).

The Spearman’s rank correlation test was performed to identify possible correlations among the prognostic indicators used in our study (Table 9). This analysis indicated a moderate negative correlation between DSR1 and TOF, and a moderate positive correlation between DSR1 and RVU. A weak negative correlation was observed between DSR1 and RVU, and weak positive correlations were observed between NOI and IT, and between RVU and LS.

**Table 9:** Correlation results for the prognostic indicators

	**Gender**		**Age**		**TOF**		**RVU**		**NOI**		**IT**		**LS**	
	***r***	***P***	***r***	***P***	***r***	***P***	***r***	***P***	***r***	***P***	***r***	***P***	***r***	***P***
**DSR1**	−0.403	0.68	−0.142	0.08	−0.590	0.00	−0.322	0.00	0.014	0.86	−0.051	0.53	−0.018	0.82
**Gender**	-	-	−0.067	0.94	−0.421	0.67	−0.258	0.79	−0.383	0.70	−0.950	0.34	−0.337	0.73
**Age**	-	-	-	-	0.172	0.48	0.182	0.02	−0.017	0.83	−0.159	0.54	0.039	0.63
**TOF**	-	-	-	-	-	-	0.584	0.00	−0.031	0.72	0.064	0.46	0.159	0.06
**RVU**	-	-	-	-	-	-	-	-	−0.139	0.09	0.073	0.38	0.300	0.00
**NOI**	-	-	-	-	-	-	-	-	-	-	0.340	0.00	0.237	0.00
**IT**	-	-	-	-	-	-	-	-	-	-	-	-	0.165	0.04

*TOF*, Time to initiate oral feeding; *RVU*, Amount of individual treatment (revenue value unit); *NOI*, Number of orotracheal intubations; *IT*, Intubation time; *LS*, Length of hospital stay; *r*, Spearman’s correlation coefficient; *P*, significance value; *DSR1*, Dysphagia severity rate 1.

## Discussion

Recently, increased regulation has required rehabilitation programs to report their results and outline the goals of the rehabilitation process effectively and efficiently. It is essential to introduce prognostic/quality indicators in order to clearly understand and manage the quality of health care. Using prognostic/quality indicators in hospital units improves the analysis of performance over time as new procedures and technology are introduced [19]. This study represents the largest group of Brazilian patients submitted to prolonged OTI who have been assessed for possible prognostic indicators related to the swallowing functional outcome at hospital discharge.

In a large group of patients submitted to prolonged OTI, we have demonstrated that, among patients who were assessed by a BSE, the ASHA NOMS level at the initial swallowing assessment (DSR1), the time to initiate oral feeding (TOF) and the amount of individual treatment (RVU) were related to a higher probability of reaching good treatment outcomes for dysphagia resolution. Among these indicators, the DSR1 is the strongest predictor. Also, the DSR1 correlated significantly with the TOF (that is the higher the ASHA NOMS level at the initial swallowing assessment the less time is needed to initiate oral feeding) and with the RVU (that is the higher the ASHA NOMS level at the initial swallowing assessment the less intervention is needed by a therapist). This finding validates the importance of the initial assessment determining the outcome of a patient with dysphagia following prolonged OTI [18].

In accordance to our results, previous researchers also found that neither age nor the duration of intubation appears to be a significant factor affecting oral intake [1,18,25,36]. The literature indicates the age variable as being implicated in the presence and resolution of the swallowing impairment [8,16,21]. However, our study suggests that the age variable did not seem to significantly interfere in the resolution of dysphagia. According to the criteria adopted in our hospital (that is public, high complexity, high rate of bed turnover), once patients reach adequate stable clinical conditions, they are discharged. In many cases, functional swallowing has not yet been reached. According to the Brazilian Health System, patients will receive speech-language pathology follow-up in specialized health care centers. For this reason 59 patients (approximately 40% of the individuals) were discharged from the hospital even though they did not reach a good dysphagia resolution. We believe that if these patients were followed until dysphagia resolution, our results would probably indicate significant differences regarding the variable age.

The association between intubation duration and severity of dysphagia is supported by the Barker *et al*. review [15] and other studies [6,15]. However, this association has not been reported in other analyses [1,8,12,37,38]. The study presented by Stauffer *et al.*[38] indicated no correlation between the duration of endotracheal intubation in intubated patients and the severity of laryngeal lesions. Many factors could account for this discrepancy, such as differences in sample size, event rate and intubation duration. Although this association is plausible based on the likely increased degree of oral, pharyngeal and laryngeal damage in patients intubated for long periods, it also remains possible that short intubation duration is sufficient to cause dysphagia [39]. The association between intubation duration and dysphagia most certainly needs to be further explored.

We would like to highlight that the result related to the time to initiate oral feeding. Based on our results, we believe that the earlier oral feeding is introduced the higher the probability of reaching good dysphagia outcomes. We also found positive results regarding the recovery of swallowing impairment until dysphagia resolution/hospital discharge. We observed that most patients presented a favorable progression of oral intake. This general pattern of improvement in the swallowing ability during the length of hospital stay is similar to the pattern of positive outcomes that have been previously reported in the specialized literature [17,18,25].

Our study also showed that poorer swallowing status at the initial swallowing assessment is a good indicator of longer swallowing management. Our findings agree with those of other studies in that preadmission functional status was also a highly relevant prognostic factor of amount of treatment (that is RVU) [14,18,40]. Previous studies suggest that a lack of accuracy in initial evaluation could impact both the dietary level assigned to patients and therefore the amount and type of treatment received [18]. The use of functional rating scales to evaluate patients with swallowing disorders has emerged over the past years [27,41-43]. Several screening methods for dysphagia have been validated [44-46]. In this study, a validated reliable clinical bedside protocol was used.

Finally, our study had several limitations. First, the results of this study have been derived from a hospital-referred cohort of patients after prolonged OTI and therefore may reflect some hospital-referral bias. Second, the conclusions drawn can only be applied to patients exhibiting some degree of dysphagia as previously discussed. Third, the clinical assessment of impaired swallowing has evident limitations and a videofluoroscopy (VFS) examination would be required for all patients. However, clinical examination, cervical auscultation and oximetry changes (that is BSE) increased the diagnostic sensitivity, and thus, the probability of identifying patients with silent aspiration [45]. Also we have to consider that although VFS is the gold standard to study oral and pharyngeal mechanisms of dysphagia and aspiration [39,47,48], it is unfeasible to perform a VFS on every patient with dysphagia (that is age, medical condition, costs and so on). A simple BSE can be used to identify patients at risk for dysphagia after prolonged OTI [6]. Third, inherent in the design of our retrospective, observational cohort study is an inability to draw conclusions about the severity of the diseases of the patients included in the study. Since patients were recruited from different ICUs of our hospital, we were unable to reach a consensus of which information could be used to characterize patients’ clinical status severity (that is each ICU uses a different protocol to determine disease severity). Similarly, some very important variables were inconsistently charted or not charted at all, thus were not available for our analysis. For example, we were unable to obtain (1) a reliable marker of sedation at the time of swallow assessment; (2) height data to calculate body mass index; (3) data on the presence of preexisting swallowing dysfunction; (4) information about endotracheal tube size. Future studies in our institution will most certainly include these variables.

Dysphagia is a major side effect of prolonged OTI. Prognostic data can be beneficial to health professionals, rehabilitative facilities providing care, insurance companies, and patients and their families. When looking at developing countries, the prolonged intensive medical and nursing care required by many patients places extra demands on a stretched health care budget [49]. Knowing the statistically significant factors that contribute to patient outcome as determined by this study reiterates the urgency for accuracy and consistency during the initial assessment within a health facility.

## Conclusions

The main contribution of the current research is related to the swallowing functional level at admission as a significant prognostic indicator of good swallowing outcome (that is ASHA NOMs level 6/7). The level of swallowing impairment, the time to initiate oral feeding and the amount of individual treatment can be used as clinical indicators to predict swallowing rehabilitation outcomes.

Given the current trend of having an evidence-based practice, studies of prognostic indicators in different populations with dysphagia can contribute to the design of more effective procedures when evaluating, treating, and monitoring individuals with this type of disorder. We believe that the measurement of prognostic indicators for swallowing rehabilitation outcomes should be routinely included in interdisciplinary hospital practice.

## Key messages

•The development of postextubation swallowing dysfunction is well documented in the literature with high prevalence in most studies, ranging from 44 to 87%.

•The results of this study suggest the swallowing functional level at admission as a significant prognostic indicator of good swallowing outcome.

•This study represents the largest group of Brazilian patients submitted to prolonged OTI who have been assessed for possible prognostic indicators related to the swallowing functional outcome at hospital discharge.

•Postextubation dysphagia persists at the time of discharge in a large portion of patients (59 (40%) of 148 patients in our study).

•When looking at developing countries, the prolonged intensive medical and nursing care required by many patients places extra demands on a stretched health care budget. Knowing the statistically significant factors that contribute to patient outcome as determined by this study reiterates the urgency for accuracy and consistency during the initial assessment within a health facility.

## Abbreviations

ASHA NOMS: American Speech-Language-Hearing Association National Outcome Measurement System; BSE: Bedside swallowing evaluation; CI: Confidence interval; DREP: Dysphagia risk evaluation protocol; DSR: Dysphagia severity rate; ICU: Intensive care unit; IT: Intubation time; LS: Length of hospital stay; NOI: Number of orotracheal intubations; OR: Odds ratio; OTI: Orotracheal intubation; RVU: Revenue value unit; SLP: Speech-language pathologist; TOF: Time to initiate oral feeding; VFS: Videofluoroscopy.

## Competing interests

The authors declare that they have no competing interests.

## Authors’ contributions

DPM contributed to data collection and analysis, to the interpretation of the results, to the manuscript writing and provided substantial scientific contribution. FCS contributed to the interpretation of the results and manuscript preparation. LDM contributed to the data collection and analysis. BZ contributed to the study analysis and manuscript preparation. CRFA conceived the study, and contributed to data analysis and manuscript preparation. All authors have read and approved the manuscript.
